# Race Differences in Allostatic Load Among Black and White Men: Does Age Matter?

**DOI:** 10.1093/geroni/igab046.1632

**Published:** 2021-12-17

**Authors:** Paul Archibald, Sarah Hill, Hossein Zare, Marino Bruce, Keith Norris, Keith Whitfield, Roland Thorpe

**Affiliations:** 1 CUNY, Staten Island, New York, United States; 2 Johns Hopkins School of Medicine, Baltimore, Maryland, United States; 3 John Hopkins Bloomberg School of Public Health, Baltimore, Maryland, United States; 4 University of Houston, Houston, Texas, United States; 5 University of California Los Angeles, Los Angeles, California, United States; 6 University of Nevada Las Vegas, Las Vegas, Nevada, United States; 7 Johns Hopkins Bloomberg School of Public Health, Baltimore, Maryland, United States

## Abstract

Although Black-White disparities in health and mortality among men persist, there has been a paucity of work focusing on race differences in physiological dysregulation of biological processes resulting from the cumulative impact of stressors among men. The purpose of this study was to assess potential race differences in Allostatic Load (AL) among adult men and if such differences varied by age. Data were drawn from the 1999-2016 NHANES and the study population included 21,529 non-Hispanic Black (NHB) and 34,282 Non-Hispanic White (NHW) born in US. Adjusting for potential confounders, NHB men 25-44 and 45-64 had a higher AL score (OR = 1.19, 95% confidence interval (CI) 1.00, 1.42) and (OR = 1.14, 95% confidence interval (CI) 1.02, 1.28) NHW men. No race differences with respect to AL score were observed among the other age groups. The results suggest that age plays a role in race differences in AL

